# Broadening the application of forensic science in an era of standardization

**DOI:** 10.1080/20961790.2022.2099338

**Published:** 2022-11-04

**Authors:** Derek Congram, Oran Finegan

**Affiliations:** International Committee of the Red Cross

In late July 2021, the Ministry of Veteran Affairs of the People’s Republic of China released a statement about the repatriation of the remains of Chinese soldiers from the Korean War, which had been recovered by the Republic of Korea:

“Following humanitarian principles and in the spirit of friendly consultation and practical cooperation, China and South Korea… will further deepen the exchanges and cooperation between the two countries in the joint search, excavation, and identification of the remains of the Chinese People’s Volunteers in South Korea” (translation to English by Yizhang Fan) [1].

This statement reflects the motivation for the introduction of forensics into the portfolio of expertise within the International Committee of the Red Cross in 2003: a humanitarian mandate and a spirit of pragmatic collaboration among countries to mitigate the lingering harm caused by armed conflict and other situations of violence, and to address the needs of affected people. These are just two examples of a broadening of forensic science application, which goes well beyond the common, narrow conception of forensic science as supporting criminal investigation. Increasingly, and especially in recent years, we are witnessing an increased socialisation and utilisation of forensic science in humanitarian work: in refugee camps, concurrent with ongoing war, at pandemic burial sites, hospital morgues, remote villages, and even in university classrooms. This evolution of thought and practice is what motivated this special issue of *Forensic Sciences Research*.

Moon [2] effectively traces forensic practice’s “departure from its regular interpretation” of criminal investigation (p. 40). However, her argument that humanitarian applications are distinct from the traditional *legal* ones, would be more accurately expressed by replacing “legal” with “judicial”. Moon’s characterization of “the shift towards humanitarianism…to challenge the historic subordination of forensics” (p. 40) to law also warrants consideration. She is correct that humanitarian applications challenge the conventional hierarchy of objectives (e.g. prioritising criminal trials of high-ranking military and political figures), but the articles in this issue demonstrate very clearly that humanitarian applications of forensic science are still very much grounded in and framed by local, national, and international law (particularly International Humanitarian Law). Therefore, the expansion of forensic science to humanitarian contexts should not be seen as “loosening forensics from its parent discipline, law” (p. 46).

Applying forensic sciences to humanitarian work includes putting the needs of principal beneficiaries at the centre and this can shift the direction of investigations so that needs are defined by the primary stakeholders. As Dahal [3] makes uncomfortably clear in this issue, existing forensic protocols might not be aligned with what victim families actually want and need.

In parallel with a broadening of application, we are seeing advances around the world in forensic science through the development of technology, protocols and standards [e.g. 4–7].

One might mistakenly believe that these two movements are working at odds with one another: standardization and accreditation implement stricter protocols that govern methods and interpretations, yet the expanded application of forensics to humanitarian work [8] broadens the contexts and activities of applied forensic science. Who would have imagined 20 years ago that the International Committee of the Red Cross—a strictly humanitarian and neutral organization—would today employ over 100 highly experienced forensic geneticists, odontologists, pathologists, anthropologists, and death investigators from around the world? Are the commonly invoked, yet difficult-to-define “universal standards” now a more complicated ideal?

Several articles in this issue touch on potential contradictions or compatibility of different objectives that can be accomplished with forensic science [9, 10]. Something that a broader perspective helps us realize is that there are contextual factors that govern *the use* of what forensic science can help us know. Forensic scientists must be mindful of this in their work: as a community we can accomplish great things and ensure high standards of documentation, analysis, and interpretation that produce precise and accurate results. However, what is done with our results will shift across time and place. The burden is therefore on the community of scientists to ensure that our work in *all* contexts is done well and can serve distinct purposes, even when there are immediate limitations (e.g. ongoing armed conflict) impacting capacity or will regarding the results of forensic analysis. The adage in archaeology is that it is a destructive process that alters a scene, objects, human remains, and their spatial relationships, so there is only one opportunity to “get it right”. The same could be said of death scene documentation, management of dead bodies in disasters, clinical forensic exams, and autopsies.

Growing standardization and accreditation are very welcome developments; however, they tend to be focused only on places where well developed forensic systems and infrastructure exist. The adoption and application of these standards more broadly will be limited as long as sizable inequalities in forensic technology and skills persist. Fortunately, however, as Congram et al. [11] note, our perception of the problem is sometimes exaggerated, premised on the idea that the latest technology is required to ensure the most precise, accurate, and quickest answers. Sometimes two measuring tapes, two spatial reference points and basic geometry can serve just as well as a state-of-the-art laser scanner (and the former does not require batteries).

This is not to say that we should be dismissive of technological developments. Many countries have memorialized a selected “Unknown Soldier”, who represents the millions of others who were not recovered and identified from past conflicts [12–14]. Today, the recovery and identification of contemporary and past dead due to warfare is increasingly feasible thanks to advances in forensic science. With a greater realisation of the role forensics can play in this domain we are now beginning to see other countries dedicate themselves to this task.

In the second issue of *Forensic Sciences Research*, Vieira and Shen [15] note that their vision for the journal is that it be a forum for the dissemination of the most recent advances in the forensic science community, but also that the journal be “an open door for disseminating the reality, anguish and thoughts of those who provide forensic services in particularly difficult circumstances and environments” (p. 1). Something invoked by this statement, and what the articles in this issue overwhelmingly show, is that if we want to effectively use forensic science to alleviate human suffering, we need to clearly understand *how* to do this. As Congram et al. [11] mention in this issue, the adage of “Do No Harm” is typically a gross simplification of reality. Exhumations and autopsies can be painful for families. Destructive sampling of victim remains might be necessary for scientific identification, but this can conflict with cultural beliefs about the dead and how they should be treated. These beliefs and values vary considerably around the world [e.g. 16, 17]. However, rather than be frustrated by such variation—something which again has implications for standardization and protocols—we should critically examine how to adapt protocols to particular contexts and specific needs.

We are excited about the geographic and thematic diversity that this special issue demonstrates. We believe that this illustrates the global resonance and recognition of the importance of forensic science being adopted by the humanitarian sector. Also impressive is that despite this breadth, geographic, and cultural variation, all articles demonstrate methods or models that promote the dignification of victims and their families: be it innovative techniques in constrained circumstances and with open-source software [18], active family and community engagement [3, 9, 11], advocating models that better demonstrate stakeholder carec, or calling for greater international collaboration on common problems [10, 19, 20]. We believe that forensic scientists do what they do because they love science. The articles in this special issue of *Forensic Sciences Research* show that what we love even more is using science to help people.


Derek Congram and Oran Finegan *International Committee of the Red Cross*
dcongram@icrc.org


